# Predictors of Friendship Skills for Adolescents with Asthma: An Analysis of Parent Report on the 2022 National Survey of Children’s Health

**DOI:** 10.3390/children12020233

**Published:** 2025-02-15

**Authors:** Brandon Workman, Laura A. Nabors, Danielle Pierre Hixon, Ashley L. Merianos, Cathleen Odar Stough, Joshua S. Bernstein, Jonathan A. Bernstein

**Affiliations:** 1 Department of Health Promotion and Education, School of Human Services, College of Education, Criminal Justice, Human Services, and Information Technology, University of Cincinnati, Cincinnati, OH 45221, USA; naborsla@ucmail.uc.edu (L.A.N.); pierredm@mail.uc.edu (D.P.H.); merianal@ucmail.uc.edu (A.L.M.); 2 Department of Psychology, College of Arts and Sciences, University of Cincinnati, Cincinnati, OH 45221, USA; odarcc@ucmail.uc.edu; 3 Department of Internal Medicine, Division of Rheumatology, Allergy and Immunology, College of Medicine, University of Cincinnati, Cincinnati, OH 45267, USA; bernstj@ucmail.uc.edu (J.S.B.); bernstja@ucmail.uc.edu (J.A.B.); 4 Bernstein Allergy Group, Cincinnati, OH 45236, USA

**Keywords:** adolescents, asthma, friendship skills, national survey of children’s health, allergies, overweight, parental coping

## Abstract

Background/Objectives: This study assesses parent reports of adolescent- and parent-level factors related to friendships among adolescents with asthma. This study serves to inform physicians and other health care professionals of factors related to difficulties making friends for adolescents with asthma, providing information from parent reports to identify risk and resilience factors related to friendships. Methods: Adolescents aged 12–17 years with current asthma from the 2022 National Survey of Children’s Health (NSCH) were selected for the study (*n* = 1812). A weighted logistic regression analysis was performed to explore predictors related to making and keeping friends. Adolescent-level predictors were asthma severity, allergies, body mass index, having behavioral problems, and adolescent sex and race/ethnicity (non-Hispanic White, and others, including Hispanic). Parent-level predictors were parent stress and coping. Results: Adolescents who were female, non-Hispanic White, had moderate/severe asthma symptoms, had current allergies, were overweight, and had behavioral problems were more likely to have difficulty making and keeping friends than adolescents who were male, non-White, had mild asthma, did not have allergies, were a normal weight or underweight, and did not have behavioral problems. Parents who reported difficulty coping with parenthood and its associated stressors were more likely to report that their adolescents had difficulty making friends. Conclusions: Pediatric moderate-to-severe asthma patients whose parents had difficulty coping with stress were more likely to exhibit greater difficulty making and keeping friends. Health care professionals managing these patients should incorporate questions into their history that address behavioral problems and parental household stress growing up to determine optimal strategies for improving friendship relationships. Whether this strategy leads to better asthma control and outcomes requires further investigation. In future studies, case studies with information about changes in friendships over time for adolescents with asthma should be carried out. The case studies could potentially highlight social strategies to use to improve friendship skills, and ultimately friendship-making for this vulnerable group.

## 1. Introduction

Understanding factors related to the friendships of adolescents with asthma assists health care professionals when they address needs for social support or when they make recommendations for youths with social difficulties [1,2,3,4,5]. Friendships offer social support to build youth resilience, and have been associated with better chronic illness control, and indicators of good health, including reduced anxiety, and more positive endocrine and immune status for youths who have asthma [1,5,6]. On the other hand, adolescents with asthma may have difficulty making and keeping friends, related to their health conditions, and as such, increasing knowledge about health and mental health problems related to friendships will provide information to improve social support for this vulnerable group [5]. It is important to examine health and psychosocial factors related to asthma in different populations to understand critical factors related to the social functioning of adolescents.

Several adolescent-level health variables may be related to friendship difficulties for adolescents with asthma. Allergic rhinitis and other comorbid allergic illnesses are a risk factor for asthma and often precede or co-occur with asthma [7]. It is estimated that between 60% and 80% of adolescents with asthma may have an allergic component, exacerbated by indoor and outdoor allergens [8,9]. Adolescents with asthma and allergies may face social isolation and report problems, such as embarrassment, that impact peer relationships leading to social difficulties [10,11,12,13]. The current study makes a unique contribution by examining whether children with asthma and allergies have difficulties with friendships. Asthma severity may also play a role as adolescents with severe asthma symptoms experience challenges in developing their social skills and adhering to multilayered treatment plans [6].

Obesity and behavioral problems may also negatively influence abilities to form and maintain friendships. Previous research has highlighted the predictive role of increased body mass index (BMI) and heightened asthma morbidity in adolescents with asthma [14,15]. Obesity may also be linked to social difficulties as adolescents with asthma who are obese face social marginalization or experience difficulties with making friends, and it is important for health professionals to assess friendships to make recommendations to improve involvement in activities with peers [16]. In addition, adolescents with asthma, compared to those without asthma, have been found to have a higher incidence of behavioral problems, such as difficulty with impulsivity, and a lack of emotional control and attentional focus, which are negatively related to social skills [17,18]. Adolescents with deficits in these areas may experience rejection by peers, further impacting friendships [18,19].

Adolescents’ friendships may be tied to parental coping and the experience of stress if parents are not available to support adolescents as they strive to spend time with and interact with peers. Previous studies have indicated that adolescents with asthma may have a parent or parents who experienced stress and difficulty with raising their adolescents [20,21]. Parental stress and difficulty coping may make it difficult for parents to assist with social issues, which could explain why many adolescents with asthma have difficulties with peer relationships [22]. Parents who have good coping skills typically are more available to provide support for their adolescents, which has been associated with improved asthma symptoms and quality of life [20,23]. Similarly, adolescents with asthma whose parents are experiencing less stress and are coping well may report higher quality of life and, therefore, be more likely to make and keep friends. By examining parental stress and coping, researchers can provide guidance about how parent functioning is related to the social functioning of adolescents, highlighting the inter-relations between parent and youth functioning.

The current study presents findings from an analysis of data from the National Survey of Children’s Health (NSCH; [24]). This study identified parent perceptions of critical child-level health and mental health predictors and examined the association of parent functioning to the friendship skills of adolescents, assessing the linkage among positive coping and reduced stress of parents and positive social functioning of adolescents. We hypothesized that adolescents with current allergies, more severe asthma, and who were overweight would have more friendship difficulties [6,7,14]. It was postulated that adolescents with asthma and behavioral problems would have more friendship difficulties than those with asthma who did not have behavioral problems [18,19]. Further, we anticipated that the sex and race/ethnicity of adolescents would impact their abilities to make and keep friends, with female adolescents and racial minorities reporting a higher level of friendship difficulties than male adolescents or adolescents who were White [4,25]. Understanding the relations among demographic factors and friendship skills provides information to inform assessments by health professionals. In addition, we expected that parents who reported more stress and more difficulty coping, as related to the demands of parenthood would have adolescents with asthma who were more likely to experience difficulties with friendship-making compared to parents reporting less stress and better coping [20,21]. Thus, the current study adds to the literature by examining relations among adolescent physical and mental health, parent coping, and adolescents’ abilities to make and maintain friendships. As such, the findings provide guidance about risk areas for health professionals to watch for, ask questions about, and provide recommendations to improve social functioning for those adolescents who might benefit from suggestions and potentially counseling to improve their friendship skills.

## 2. Materials and Methods

### 2.1. Participants and Procedures

Data for this study were from the 2022 NSCH—a cross-sectional survey delivered online or via mail in states and territories of the United States (U.S.) conducted by the U.S. Census Bureau [26,27]. Information is gathered about one child (under 18 years of age) from caregivers (referred to as parents throughout this manuscript) as respondents. Questions address different health topics, including child physical and mental health, as well as child and parent functioning. Information about the 2022 NSCH methodology is available from the U.S. Census Bureau [27].

Participants were caregivers (parents) of 1812 adolescents between the ages of 12 and 17 years who had current asthma. We excluded 15,932 (84.5%) adolescents aged 12–17 years who did not have asthma and 1107 (5.9%) adolescents who had been diagnosed with asthma in their lifetime but did not have it currently according to parent reports. This study was deemed as non-human subjects by the Institutional Review Board at the University of Cincinnati.

### 2.2. Instrumentation

We used SPSS codebook for the NSCH [28]. The variable used to assess asthma status was “asthma_22”, which inquired if the child has asthma currently (current asthma, according to a doctor or health care professional), was told previously the child had asthma but the child does not have asthma currently, or the child never had asthma. The outcome variable used to assess friendship difficulties was based on survey responses if the adolescent had either no difficulty making or keeping friends or “a little” and “a lot” of difficulty making or keeping friends (“MakeFriend_22”).

**Parent Reports on Adolescent-Level Variables**. We recoded the following health variables: (a) current allergies, (allergies_curr: “Has a doctor or other health care provider ever told you that this child has allergies?”), 0 = no, 1 = yes; (b) asthma severity, (asthmasev_22 for those with current asthma), 0 = mild, 1 = moderate/severe; (c) BMI (BMI4_22: body mass index for child BMI-for-age was recoded into two categories), 0 = less than the 85th percentile; and 1 = 85th percentile and higher; and (d) current behavioral problems (behavior_22: “Has a doctor, other health care provider, or educator ever told you that this child has behavioral or conduct problems?”), 0 = no, 1 = yes. Adolescent-level demographic variables were recoded: (a) sex (sex_22), 0 = male, 1 = female; and (b) race/ethnicity (race4_22: Hispanic, White non-Hispanic, Black non-Hispanic, and Multiracial non-Hispanic), 1 = White non-Hispanic, 0 = Black non-Hispanic, Hispanic, and Multiracial non-Hispanic.

**Parent Reports of Their Coping and Stress**. We recoded parenting variables: (a) parental coping (parcoping_22: “How well do you think you are handling the day-to-day demands of raising children?”), 0 = coping very well/coping somewhat well and 1 = not well (“not very well/not coping well at all”); and (b) parental stress was analyzed by combining responses to three questions (paraggrav_22: “During the past month, how often have you felt: (1) that this child was much harder to care for than most children his or her age?; (2) that this child does things that really bother you a lot?; and (3) angry with this child?”), which were recoded as 0 = seldom feels stress from parenting and 1 = usually or always feels stress from parenting.

### 2.3. Data Analyses

Descriptive statistics for adolescents with current asthma were conducted using data for adolescent-level predictors, and parent stress and coping predictors (raw counts, weighted percentages, and chi-square tests) if parents responded to the making and keeping friends’ question. A weighted logistic regression analysis (with the FWC weighting variable for the NSCH, 28) was used to examine relations among predictor variables (adolescent-level variables, and parent stress and coping) and making and keeping friends (outcome variable) for adolescents with current asthma. All data analyses were performed using IBM SPSS software Version 29 (IBM Corp., Armonk, NY, USA).

## 3. Results

Of the 1812 adolescents with current asthma, 936 (51.70%) were male and 876 (48.30%) were female. In terms of racial/ethnic groups, 1053 (58.10%) were non-Hispanic White, and 759 (41.90%) were reported as other racial/ethnic groups (Hispanic, Black, and other race groups).

Table 1 presents information on raw counts and weighted percentages for predictors and data on weighted chi-square tests examining relations among predictor variables and making and keeping friends (outcome variable).

The omnibus chi-square test was significant (*p* < 0.001), and all predictors were significantly related to making and keeping friends (*p* < 0.001; Table 2).

**Parent Reports on Adolescent-Level Variables**. All health and mental health variables were associated with friendship difficulties for adolescents with asthma. Specifically, results showed that adolescents with current allergies were 4.71 times more likely to have difficulty making and keeping friends than those without concurrent allergies. Adolescents with moderate/severe asthma symptoms were about 1.19 times more likely to have difficulties with friends than those with mild asthma symptoms. Overweight adolescents (BMI in the 85th percentile and higher) were about 1.40 times more likely to have difficulties with friendships. Adolescents with current behavioral problems were 4.65 times more likely to have difficulties with friendships. Sex and racial/ethnic group were also significant. Female adolescents were 1.32 times more likely to report friendship difficulties than male adolescents. Lastly, adolescents who were non-Hispanic White were 1.28 times more likely to report friendship difficulties than adolescents in other racial/ethnic groups (see Table 2).

**Parent Reports of Their Coping and Stress**. The two parent-level variables were significant in the weighted model. Parental coping difficulties led to a 5.72 times increased likelihood of reporting their adolescent had difficulty making and keeping friends. Parental stress contributed to 3.26 increased odds of reporting that their adolescent had difficulty making friends (see Table 2).

## 4. Discussion

Our findings revealed that adolescents with poorly controlled asthma, allergies, and behavioral problems, who were overweight, and had parents reporting higher stress levels increased the odds of having more difficulties making and keeping friends. Our findings, based on parental reports, were consistent with those of previous research [4,5], suggesting adolescents with asthma experience physical and psychosocial stressors that may influence their ability to make friends [3,5,29]. Study findings add to research by verifying that parent coping and child mental health and physical factors, including having allergies plus more severe asthma, are related to friendships. As such, the findings provide guideposts for health professionals assessing the social functioning of adolescents who have asthma. Importantly, having moderate to severe versus mild asthma symptoms and having allergies were related to difficulty in making and keeping friends in the current study [11,12,13]. It may be that adolescents with more severe upper and lower respiratory symptoms face activity limitations that prohibit them from participating in activities with peers. The impact of exercise-induced limitations from allergic respiratory disease on social interactions warrants further investigation [10].

Adolescents with asthma who were overweight (BMI of 85th percentile and higher) reported more difficulty with friendships than adolescents who were either a healthy weight or underweight (BMI less than 85th percentile). Poor asthma prognosis and being overweight may result in social marginalization [15,16,30]. Activity limitations [31] or stigma related to weight [32] have been factors that affect the friendships of adolescents who are overweight. It will remain important for physicians and other health care professionals to reinforce sustainable, non-surgical, non-pharmacologic approaches to asthma and comorbid overweight/obesity, including tailored diet and exercise plans [33,34]. Encouraging involvement in physical activity, such as increasing walking and involvement in sports (e.g., at least 60 min one day per week), may be an avenue for adolescents to improve BMI, asthma prognosis, and ultimately social engagement [31,34].

Behavioral problems were related to friendship difficulties. Awareness of potential social difficulties for young people with asthma is critical for screening purposes. Similarly, being female was an additional comorbid risk factor for making friends; however, the general nature of the questions in the data set did not allow for detailed exploration as to why this was the case. Given the limited research in this area, it will be beneficial to conduct further qualitative studies to explore female adolescents’ reasons for reporting difficulties with engaging with peers and maintaining friendships [25]. Adolescents who were White had more difficulty making friends than adolescents of color [4,5]. Hence, our hypothesis predicting adolescents of “other” racial groups having more difficulty making friends was not supported. Continuing to assess demographic factors, developing social support networks, and recognizing the need for mental health referrals will be necessary to further address adolescent friendships [4].

Parental stress and the ability to cope with the demands of parenthood showed expected relationships with friendships. If health care professionals learn of parental stress (e.g., aggravation with the parenting role), they may wish to inquire about potential adolescent vulnerabilities when it comes to making and keeping friends. Promoting parent coping often enhances family well-being [20,23]. Learning coping skills and parenting strategies may reduce parental stress, fostering an environment for adolescents to imprint social resiliency and have improved abilities to make and keep friends [20,23]. Assisting parents to determine ways to cope with the stress of managing adolescent asthma and providing them with ideas to help their adolescents improve their self-management of asthma may relieve burdens of care.

**Limitations**. This study is cross-sectional and retrospective. Friendships may change over the course of adolescence [35]. The variable for allergies was vague and did not differentiate between environmental or perennial allergic exposures. For example, if a person with asthma has a pet allergy and is unable to go over to a friend’s house, this is valuable information to further ascertain and may be a good opportunity to initiate allergen immunotherapy. Parents provided data for the NSCH, and adolescents’ perceptions of their abilities to make and keep friends may be substantially different than those of their parents. The large sample size and multiple analyses may have increased the chance of a type I error. Results highlight relationships among critical health and mental health factors for adolescents and friendships and show associations among parent functioning and friendships; however, our analysis does not explain the mechanisms behind these associations. Moreover, the general nature of the questions in the NSCH do not explore underlying mechanisms that account for the relations among our predictors and friendships. Therefore, research assessing adolescent perspectives of friendships, and health and social barriers, and facilitators of friendships may provide adolescent views about what is needed to facilitate friendship-making. Future qualitative and/or longitudinal studies should examine adolescents’ views of their social lives and may also elucidate the mechanisms accounting for the relations between physical health and mental health factors as well as parent functioning and friendships for adolescents with asthma.

## 5. Conclusions

We have identified risk factors, based on parent reports, among adolescents with asthma, which negatively impact the ability to make and maintain friendships. Some key risk factors for a lack of peer support included being female, having a BMI in the 85th percentile and higher, having current allergies, and experiencing more severe asthma symptoms. Results provide ideas for health care professionals to determine risk factors to assist in the examination of peer support and need for intervention for adolescents who have difficulty with friendships. In our view, teachers and after-school activities are important contexts for friendships. As such, communication between health care professionals and teachers about factors impacting social relationships can support adolescents. Understanding the relations among academic performance and friendships and peer support is an area for continued study. Furthermore, when risk factors are present (e.g., health conditions such as allergies or being overweight, or behavioral difficulties), inquiring about social support as well as involvement in extracurricular activities and hobbies may spur ideas to enhance the development and maintenance of friendships [36]. Furthermore, identifying parental stress and challenges with coping with the demands of parenting an adolescent with asthma, and whether this impacts parent abilities to support the development of peer relationships should be evaluated in cases where adolescents report not having strong or supportive friendships. Health care professionals and researchers should continue to assess challenges with friendships and explore new interventions for cultivating social skills among adolescents with asthma. By addressing risk factors negatively impacting abilities to make and keep friends for adolescents who have asthma, it is possible to improve the overall disease burden in this vulnerable pediatric population.

## Figures and Tables

**Table 1:** Raw counts and percentages as well as weighted chi-square *p* values for adolescent and parent variables for making and keeping friends.

Predictor/Categories	Raw Counts and Weighted Percentages	*p* Values
**Adolescent Current Allergies**		<0.001
No	81 (6.80%)	
Yes	1225 (93.20%)	
**Adolescent Asthma Severity**		<0.001
Mild	907 (68.30%)	
Moderate/Severe	399 (31.70%)	
**Adolescent BMI Status**		<0.001
Underweight/Normal (<85th percentile)	862 (57.90%)	
Overweight/Obese (≥85th percentile)	444 (42.10%)	
**Adolescent Current Behavioral Problems**		<0.001
No	1148 (87.60%)	
Yes	158 (12.40%)	
**Parent Stress**		<0.001
No	1198 (89.80%)	
Yes	108 (10.20%%)	
**Parent Coping**		<0.001
Coping well or somewhat well	1277 (97.20%)	
Not coping well	29 (2.80%)	
**Adolescent Sex**		<0.001
Male	706 (49.10%)	
Female	600 (50.90%)	
**Adolescent Race/Ethnicity**		<0.001
Other	536 (44.20%)	
White, non-Hispanic	770 (55.80%)	

Notes. There were 1306 adolescents with current asthma whose parents responded to questions for all variables in the model. The weighting variable for the NSCH (FWC) was used in chi-square tests comparing predictors to the outcome variable. Table 2 presents the odds ratios and confidence intervals for the weighted logistic regression model.

**Table 2:** Weighted logistic regression model for adolescent and parent predictors in making and keeping friends.

Predictor/Categories	Adjusted Odds Ratio	95% Confidence Interval	
		**Lower**	**Upper**
**Adolescent Current Allergies**	4.71	4.61	4.81
No			
Yes			
**Adolescent Asthma Severity**	1.19	1.19	1.20
Mild			
Moderate/Severe			
**Adolescent BMI Status**	1.40	1.385	1.405
Underweight/Normal (<85th percentile)			
Overweight/Obese (≥85th percentile)			
**Adolescent Current Behavioral Problems**	4.65	4.59	4.70
No			
Yes			
**Parent Stress**	3.26	3.22	3.31
No			
Yes			
**Parent Coping**	5.72	5.57	5.86
Coping well or somewhat well			
Not coping well			
**Adolescent Sex**	1.32	1.31	1.33
Male			
Female			
**Adolescent Race/Ethnicity**	1.28	1.27	1.29
Other			
White, non-Hispanic			

**Notes**. There were 1306 adolescents with current asthma whose parents responded to questions for all variables in the model. The weighting variable was FWC. All predictors were significant at *p* < 0.001.

## Data Availability

Data for this study were from the 2022 National Survey of Children’s Health (https://www.childhealthdata.org/, accessed on 20 May 2024).
